# Happy New Year!

**DOI:** 10.1055/s-0038-1677005

**Published:** 2019-01-02

**Authors:** Elinor Switzer

**Affiliations:** 1 Project Manager Journals, Thieme Publishers, Stuttgart, Germany

Dear colleagues and friends,

Happy New Year 2019! We are pleased to share that *TH Open* , the open access companion journal to *Thrombosis and Haemostasis* , is growing well, publishing more than 65 peer-reviewed manuscripts in both clinical studies and basic science since its launch in 2017. 1

The length and breadth of articles merit some discussion, and we are pleased to highlight a small selection of the most popular articles. Amongst the clinical studies published in the journal, two papers from the RIETE Registry dealing with cancer-associated thrombosis stand out, which have been widely cited. 2 3 In the basic science field, a paper by Kashiwagi et al indicating that nicotine- and tar-free cigarette smoke extract (CSE) inhibits COX-1 activity and thereby decreases TXA _2_ production in platelets, leading to inhibition of platelet aggregation. 4

Please check out the *TH Open* homepage: www.thieme.com/THO where you can read these and all other articles for free, find all info about the journal, and access the submission site.

Manuscripts come in both as direct submissions as well as transfers from *Thrombosis and Haemostasis* . Authors benefit from rigorous peer-review, rapid online publication, as well as its unique “Pay What You Want” policy which allows authors to decide how much worth they place into the publication process and give a chance to those who are deterred by high publishing prices to submit Open Access.

As a new journal, *TH Open* is currently under application for indexing in PubMed Central. A few articles are already online on PMC. 5 6 7 8 Once we get fully accepted, all published content will be retroactively included. Further applications to other indexes will follow PMC inclusion. In the meantime, the journal is fully accessible and searchable on www.thieme-connect.com , and we will be actively promoting and highlighting articles from the journal also on Facebook.

A warm “Thank you” goes out to all those who served as reviewers for the journal as well as the dedicated Editorial Board serving *TH Open* in the past year: Marie Lordkipanidze, Alma Zernecke, Marco Proietti, Sandip Kanse, and Alfonso Tafur.

We also thank our Editorial “God-fathers” (the Editors-in-Chief of *Thrombosis and Haemostasis* ), Gregory Y. H. Lip and Christian Weber, who oversaw our launch 1 9 and have been supportive of our efforts in enhancing the journal and its content.

With best regards and warm wishes for a healthy and successful year.
